# The Role of Forkhead Box Protein M1 in Breast Cancer Progression and Resistance to Therapy

**DOI:** 10.1155/2016/9768183

**Published:** 2016-01-31

**Authors:** Raya Saba, Alhareth Alsayed, James P. Zacny, Arkadiusz Z. Dudek

**Affiliations:** ^1^Presence Saint Joseph Hospital, 2900 North Lakeshore Drive, Chicago, IL 60657, USA; ^2^University of Illinois Hospital and Health Sciences System, UI Cancer Center, Medical Center Administration Building (M/C 700), 914 South Wood Street, Chicago, IL 60612, USA

## Abstract

The Forkhead box M1 (FOXM1) is a transcription factor that has been implicated in normal cell growth and proliferation through control of cell cycle transition and mitotic spindle. It is implicated in carcinogenesis of various malignancies where it is activated by either amplification, increased stability, enhanced transcription, dysfunction of regulatory pathways, or activation of PI3K/AKT, epidermal growth factor receptor, Raf/MEK/MAPK, and Hedgehog pathways. This review describes the role of FOXM1 in breast cancer. This includes how FOXM1 impacts on different subtypes of breast cancer, that is, luminal/estrogen receptor positive (ER+), expressing human epidermal growth factor receptor 2 (HER2), basal-like breast cancer (BBC), and triple negative breast cancer (TNBC). The review also describes different tested preclinical therapeutic strategies targeting FOXM1. Developing clinically applicable therapies that specifically inhibit FOXM1 activity is a logical next step in biomarker-driven approaches against breast cancer but will not be without its challenges due to the unique properties of this transcription factor.

## 1. Introduction

The Forkhead box family is a group of over 50 mammalian proteins that is characterized by a winged helix DNA-binding domain [1]. Forkhead box M1 (FOXM1) is a transcription factor that has been well studied and implicated in normal cell growth as well as carcinogenesis. A growing body of evidence suggests a role for FOXM1 in various malignancies including breast [2], liver [3], lung [4], prostate [5], and colorectal cancer [6]. More importantly, different pathways have been elucidated but the exact mechanism remains unclear as multiple transcription factors and downstream regulatory genes are being discovered [7].

The focus of this review will be on the correlation between FOXM1 and (1) hormone and growth factor receptors pathways associated with breast cancer and (2) resistance to breast cancer therapies. We will first briefly discuss the structure of FOXM1 and its role in both normal cell functioning and carcinogenesis.

## 2. FoxM1 Structure

Fox proteins share a DNA-binding domain, the Forkhead box or winged helix domain (WHD); it consists of three helices, three sheets, and two loops or wings forming a helix-turn helix-like motif. Structural variations occur between the second and third helices, whereas the helices and sheets are highly conserved. DNA binding occurs primarily through the third helix, or recognition helix, and the second wing, which bind to the major groove and minor groove of the DNA, respectively [8].

FOXM1 protein binds the core consensus sequence (A/C)AAACAAAC [9] and, in some instances, requires interaction with other factors for DNA-binding and transcriptional activity [10]. Additionally, FOXM1 may also recruit cyclin-dependent kinase (CDK) cyclin complexes through the LXL motif. Overall, there is a multitude of factors that play a role in the transcription and target gene expression of Fox proteins, including histone deacetylase (HDAC) and RUNX [11].

The FOXM1 gene is located on the chromosomal band 12p13 [12]. It consists of seven (I–VII) exons plus two alternatively expressed exons (A1 and A2) generating three alternatively spliced coding isoforms: FOXM1a, FOXM1b, and FOXM1c. Exon A2 has been shown to be a transcriptional repressor, whereas exon A1 does not have any apparent transcriptional significance. FOXM1b is lacking exons A1 and A2 sequences [9].

## 3. Role of FOXM1 in Normal Cell Functioning and in Carcinogenesis

### 3.1. FOXM1 in Normal Cells

Studies have shown that FoxM1 exerts its effect through regulation of the cell cycle transition points. It peaks at G1/S and G2/M phases and is essential for mitosis through promotion of M phase entry [9, 13]. FOXM1 is located in the cytoplasm at late G1 and S phases [14]. It is activated by mammalian mitotic kinase Polo-like kinase-1 (Plk1) through binding to carboxyl terminal domain of FOXM1 and through further phosphorylation of two residues on this domain by cyclin-dependent kinase 1 (Cdk1) [15], cyclin E-CDK2 [16], and Raf-MEK-ERK-mediated phosphorylation [14]. FoxM1 has been shown to regulate the transcription of genes involved in cell cycle by binding to promoter of cyclin D1 and cyclin B1 genes [17] and by direct activation of transcription of the Cdc25B phosphatase promoter region [18]. In the hepatoblasts of Foxm1b −/− murine embryos mitosis was associated with decreased expression of the Aurora B kinase and Polo-like kinase 1 (Plk1) and Cdc25A phosphatase [19]. Wang et al. showed, using quantitative chromatin immunoprecipitation and expression, that FoxM1 is necessary for transcription of the Aurora B kinase, survivin, centromere protein A (CENPA), and CENPB [20]. Other mechanisms of action include upregulation of transcription of S phase kinase-associated protein 2 (SKP2) and cyclin kinase subunit 1 (CKS1), subunits of the SKP1-Cullin 1-F-box ubiquitin ligase complex, leading to degradation of p27 and p21 [20].* In vitro* experiments in mice detected FoxM1 in all proliferating cells, both adult and embryonic; once the cell had differentiated, it was noted that the expression of FoxM1 had significantly decreased [21]. The significance of FoxM1 in normal cell growth is supported by Krupczak-Hollis et al. [19] and Kim et al. [22], who showed that absence of FoxM1 in embryonic cells led to major developmental defects in the liver and lung and was ultimately fatal. Wonsey et al. [23] supported this notion by demonstrating that the loss of FoxM1 led to genetic mitotic spindle defects, delayed cells in mitosis, and induced mitotic catastrophe. FoxM1 transcriptional activity is controlled by the Small Ubiquitin-Like Modifier-2 (SUMO-2) protein. This modification of FoxM1 peaks during G2 and M phase. SUMOylation blocks the dimerization of FoxM1, thereby alleviating FoxM1 autorepression [24].

### 3.2. FOXM1 in Carcinogenesis

The first evidence of a correlation between FOXM1 and human cancer was reported by Teh et al. in 2002 [25]. Their data revealed a high level of FOXM1 expression in human basal cell carcinomas, as a downstream target of the oncogenic transcription factor, glioma-associated oncogene homolog 1 (GLI1). Halasi and Gartel [26] suggested several mechanisms that would explain the increased expression and activity of FoxM1 in cancer: (1) FoxM1 locus amplification as seen in both solid [27] and hematologic malignancies [28], (2) a high level of stability or expression of FoxM1 in cancer cells initiated through different pathways, (3) enhanced transcription of FoxM1 through promoter binding of various factors such as E2 transcription factor (E2F), c-Myc, and hypoxia-inducible factor- (HIF-) 1, (4) mutations of the tumor suppressor p53 [29, 30], and (5) activation of FoxM1 by oncogenic signaling pathways such as PI3/Akt, epidermal growth factor receptor (EGFR), Raf/MEK/MAPK, and Hedgehog. Recently Wei et al. [31] demonstrated direct activation by FoxM1 of SNAIL gene, a key regulator of epithelial-mesenchymal transition (EMT), and showed a direct correlation with expressions of FoxM1 and Snail transcription factor in human lung adenocarcinoma tissues.

**Figure 1:**
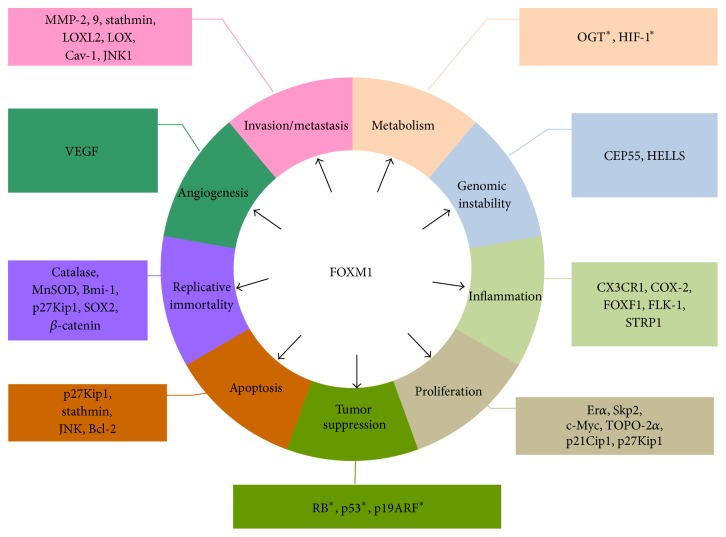
Schematic diagram of the biological role of FOXM1 in carcinogenesis. Recent research has shown that FOXM1 has a complex and diverse role in carcinogenesis. The effects of FOXM1 are mediated by numerous downstream targets as shown in this figure. In addition, several effects of FOXM1 on carcinogenesis are mediated by upstream regulators of FOXM1 (^*∗*^). Bcl-2: B-cell lymphoma/leukemia-2 protein; Bmi-1: B lymphoma Mo-MLV insertion region 1 homolog; Cav-1: caveolin-1; CEP55: centrosomal protein 55 kDa; c-Myc: myelocytomatosis viral oncogene homolog; COX-2: cyclooxygenase-2; CX3CR1: chemokine receptor CX3CR1; ER*α*: estrogen receptor alpha; FLK-1: fetal liver kinase-1; FOXF1: Forkhead box protein F1; FOXM1: human Forkhead box M1; HELLS: lymphoid specific helicase; HIF-1: hypoxia-inducible factor-1; JNK1: C-Jun NH_2_-terminal kinase-1; LOX: lysyl oxidase; LOXL2: lysyl oxidase homolog 2; MMP-2: matrix metallopeptidase-2; MMP-9: matrix metallopeptidase-9; MnSOD: manganese superoxide dismutase; OGT:* O*-linked *β*-*N*-acetylglucosamine transferase; p19ARF: p19 alternate reading frame; p21^Cip1^: cyclin-dependent kinase inhibitor 1; p27^Kip1^: p27 kinase inhibitor protein 1; RB: retinoblastoma; Skp2: S phase kinase-associated protein 2; SOX2: Sry-related HMG box2; STRP1: short tandem repeat polymorphism 1; TOPO-2*α*: topoisomerase-2 alpha; VEGF: vascular endothelial growth factor.

The development of metastatic disease is one of the essential hallmarks of carcinogenesis [32]. Studies have shown that FoxM1 plays a critical role in not only tumor metastasis, EMT, cell motility, invasion, and premetastatic niche formation [33] but other key cancer hallmarks as well (Figure 1)—reprogramming of energy metabolism; promotion of genomic instability [34, 35], inflammation, cell proliferation, and angiogenesis; evasion of growth/tumor suppression; circumvention of apoptosis; and enabling of replicative mortality [29].

## 4. Role of FOXM1 in Breast Cancer

### 4.1. FoxM1 in the Biology of Breast Cancer

The effect of FOXM1 on biology of breast cancer was evaluated in 236 women with breast cancer. Expression of FOXM1 was associated with larger tumor size, lymphovascular invasion, lymph nodes metastases, and higher stage of breast cancer. There was no significant impact of FoxM1 expression on survival when all breast cancer histologies were analyzed; however, in a subgroup of patients with estrogen receptor (ER) positive tumors, low FOXM1 expression correlated with better survival (hazard [low versus high] = 7.304, 95% confidence interval [0.897–59.45], *p* = 0.063) [36]. In another study of 501 ER positive tumors, FOXM1 was overexpressed in 20% of tumors that correlated with higher recurrence rate and shorter survival (*p* = 0.03), contributed to association of FOXM1 with stem-cell like population, increased expression of markers of EMT, and increased resistance to tamoxifen therapy [37].

**Figure 2:**
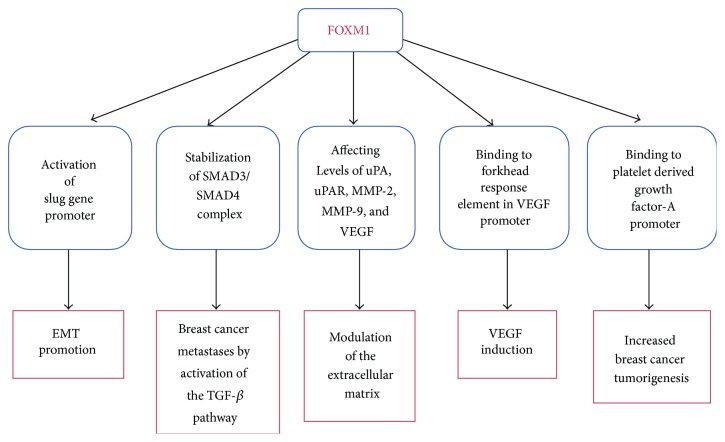
Schematic diagram of the biological role of FOXM1 in breast cancer tumorigenesis. Recent research has shown that the effects of FOXM1 on breast cancer tumorigenesis are mediated by a number of diverse biological mechanisms. EMT: epithelial-mesenchymal transition; TGF-*β*: transforming growth factor-beta; uPA: urokinase; uPAR: urokinase receptor; MMP-2: matrix metallopeptidase-2; MMP-9: matrix metallopeptidase-9; VEGF: vascular endothelial growth factor.

The complex and diverse ways in which FOXM1 promotes breast cancer tumorigenesis are depicted in Figure 2. Yang et al. demonstrated that FOXM1 promoted EMT in breast cancer by binding and activation of the promoter of SLUG gene [38]. Xue et al. showed that FOXM1 promotes breast cancer metastases by activation of the TGF-*β* pathway through interaction with SMAD3 (this prevented E3 ubiquitin-protein ligase transcriptional intermediary factor 1 *γ* [TIF1 *γ*] binding to SMAD3 and protected SMAD4 from ubiquitination) that leads to stabilization of the SMAD3/SMAD4 complex [39]. FOXM1 also has a role in modulation of the extracellular matrix by affecting levels of uPA, uPAR, MMP-2, MMP-9, and VEGF [40]. The mechanism of VEGF induction by FOXM1 was elucidated by Karadedou et al., in which they demonstrated that FOXM1 binds to Forkhead response element in VEGF promoter [41]. Yu et al. showed that binding of FOXM1 to platelet derived growth factor-A promoter led to activation of the AKT pathway and increased breast cancer tumorigenesis [42]. Nestal de Moraes et al. [43] have shown that FOXM1 upregulates antiapoptotic genes XIAP and survivin by interacting with their promoters, contributing to chemoresistance of breast cancer cells to docetaxel, paclitaxel, and epirubicin. Moreover, coexpression of FOXM1, survivin, and nuclear XIAP was associated with poor outcomes of women with stage III breast cancer with significantly reduced 5- and 10-year survival rates versus women with tumors without these features.

Breast cancer encompasses a heterogeneous group of entities that vary greatly in terms of histology, therapy, and prognosis. This notion was suggested by Sørlie et al. [44] and later confirmed in the cancer genome atlas published in 2012 [45]. Human breast malignancies can be divided into five subtypes: normal breast-like, luminal A, luminal B, HER2/Neu-enriched, and basal-like breast cancer (BBC).

### 4.2. FOXM1 and Luminal/Estrogen Receptor Positive (ER+) Breast Cancer

The effect of estrogen on both normal and malignant breast tissue is mediated through two types of estrogen receptors (ER): ER*α* and ER*β*. Although they bind to estrogen with equal affinity, ER*α* and ER*β* respond differently to estrogen stimulation: activated ER*α* induces breast epithelium proliferation, whereas ER*β* has antiproliferative and proapoptotic effects [46]. Approximately 70% of breast cancers are estrogen receptor positive (ER+) and these tumors are often better differentiated, grow slowly, and have a favorable prognosis [47].

Early research about the Forkhead box family and growth factors in breast cancer emerged in 2000. Jackson et al. provided the first evidence of a link between epidermal growth factor (EGF) and a Forkhead box family member, FOXO3a, known at that time as FKHR [48]. Influenced by these findings, Madureira et al. [49] and Karadedou [50] found a positive correlation between ER*α* and FOXM1 and an inverse correlation between ER*α* and FOXO3a. Their results showed that FOXM1 is a physiologic regulator of ER*α* expression in breast cancer cells, both at the protein and at mRNA levels. Consistent with these findings, later studies confirmed the tumor suppressive qualities of FOXO3a [51], including cases of ER+ breast malignancies [52]. Carr et al. reported transcriptional repressor function of FOXM1 inhibiting differentiation of luminal progenitor cells by inducing methylation of promoter of the zinc finger transcription factor GATA-3 through association with DNMT3b [53]. Millour et al. supported the critical role of FOXM1 in mitogenic functions of ER*α* and estrogen in breast malignancies [54]. Additionally, they showed that FOXM1 deregulation may also contribute to antiestrogen insensitivity, opening the door to practical applications of these findings in the field of therapeutics. Whereas the focus of these studies was on ER*α*, Horimoto et al. studied the effects of ER*β* expression on breast cancer [55]. Their investigations showed that ER*β* had an antiproliferative effect through repression of FOXM1 expression in breast cancer cells; this effect was mediated by an estrogen-response element within the proximal promoter region that is also a target of ER*α*.

The correlation between FOXM1 and ER+ breast cancer has also been shown on the genetic level. Ahn et al. created a 70-gene signature which was found to be of prognostic value in ER+ breast cancer patients in that FOXM1 suggested poorer prognosis [56]. This signature was found to be most helpful in cases of intermediate Oncotype recurrence scores. Other studies included genome-wide mapping by Sanders et al. which revealed a direct relationship between FOXM1 and ER*α* [57]. Their analysis uncovered another signature of 38 FOXM1-regulated genes with a prognostic value.

### 4.3. FOXM1 and Human Epidermal Growth Factor Receptor 2 (HER2)

The human epidermal growth factor receptor 2 (HER2) is a transmembrane tyrosine kinase receptor and a member of the epidermal growth factor (EGF) family [58]. The amplification of HER2 can be seen in up to 25% of breast cancer cases and has been shown to correlate with high relapse rates and poor survival [59]. In 2008, Bektas et al. provided the first evidence of a positive correlation between HER2 status and FOXM1 expression in breast cancer specimens, in comparison to normal breast tissue [60]. Additionally, their work verified the overexpression of FOXM1 in breast cancer on both the RNA and protein level. Francis et al. elaborated on the concept further through various* in vitro* and* in vivo* experiments [61]. Their work suggested that FOXM1 may in fact be a downstream target and marker of HER2 overexpression in breast cancer. This study also had translational implications by showing a suppressive role for lapatinib (a HER2 inhibitor) on FOXM1 expression at the protein, mRNA, and gene promoter levels. Subsequent data showed a role for FOXM1 in the development of trastuzumab (a HER2 antibody) resistance [62]. This is particularly important because trastuzumab resistance may develop quickly and represents a challenge in the treatment of breast cancer.

### 4.4. FOXM1, Basal-Like Breast Cancer (BBC), and Triple Negative Breast Cancer (TNBC)

Basal-like breast cancer (BBC) is an aggressive phenotype of breast malignancies that is often associated with poor prognosis [63]. BBC cells lack hormone estrogen receptors (ER) and progesterone receptors (PR) and express genes that are usually seen in basal or myoepithelial cells of normal breast tissue. Triple negative breast cancers (TNBC), on the other hand, are tumors that lack HER2 in addition to ER and PR. Although differences between these two types of breast cancer have been demonstrated, BBC and TNBC overlap significantly [64]. These tumors represent a therapeutic challenge because of lack of effective therapy. Although TNBC accounts for only 15% of breast cancer subtypes, it causes 25% of breast cancer-related deaths due to its aggressive and refractory nature [65].

EMT has been correlated with TNBC [66], and NF-kappaB is a transcription factor that has been shown to be essential in EMT in breast cancer [67]. Arora et al. incorporated these two concepts in their search for a therapeutic agent for TNBC [68]. They discovered that panepoxydone (PP), a NF-kappaB inhibitor, halted proliferation, induced apoptosis, and reversed EMT in breast cancer, in particular TNBC. Interestingly, their work revealed a downregulatory effect of PP on FOXM1 as well. A whole genome and transcriptome sequencing of TNBC cells by Craig et al. revealed consistent overexpression of FOXM1 in TNBC [69]. The role of FOXM1 has also been seen in BBC cancers, including FOXM1-dependent overexpression of MELK, a novel oncogenic kinase [70].

## 5. FOXM1 and Breast Cancer Therapeutics 

Taking into consideration the significant role of FOXM1 in breast cancer biology this transcription factor could become an attractive target for cancer treatment. Herein, we describe some breast cancer therapeutic strategies targeting FOXM1.

A FOXM1-specific small interfering RNA (siRNA) was found to be effective in reducing the expression of FOXM1 proteins* in vivo* [71]. Treatment of breast cancer cells with adenoviral vector expressing short hairpin downregulating FOXM1 led to inhibition of breast cancer tumor formation [72]. Overexpression of microRNA miR802 led to downregulation of FOXM1 and inhibited proliferation of breast cancer cells [73]. A thiazole ring containing thiostrepton, an antibiotic with antitumor activities [74], was shown to induce arrest and death of breast cancer cells through downregulating FOXM1 expression [75]. As stated earlier [24], SUMO is a posttranslational modifier that is essential for activation of FOXM1. A SUMOylation protease, sentrin-specific protease 2 (SENP2), significantly decreased SUMOylation of FOXM1 and interfered with its function [76]. Casticin, an active ingredient derived from* Fructus Viticis*, the fruit of a traditional Chinese medicine, has anticarcinogenic activity in breast cancer [77]. Recent experiments showed that it can induce apoptosis of breast cancer cells by reducing the expression of FOXM1 [78].

FOXM1 has been shown to play a critical role in development of resistance to breast cancer therapeutics. FOXM1 is a downstream target of 14-3-3*ζ*, a marker of endocrine therapy resistance in breast cancer malignancy [79]. FOXM1 was found to contribute to cisplatin (a platinum agent) resistance in breast cancer cells. The effect was thought to be mediated by the enhancement of DNA-damage repair pathways and the promotion of cell cycle progression or inhibition of cell cycle checkpoints and apoptosis [80]. FOXM1 overexpression was implicated in the resistance to trastuzumab (a HER2 monoclonal antibody) and paclitaxel (a microtubule stabilizing agent). Treatment with a siRNA targeting FOXM1 or an alternate reading frame- (ARF-) derived peptide resulted in improved therapeutic sensitivity to these agents [81]. FOXM1 caused doxorubicin resistance in breast cancer by enhancing DNA repair. The nuclear factor NF-kappa-B1 (NF*κ*B1) interacted with FOXM1 in the presence of doxorubicin to protect breast cancer cells from DNA damage [82]. Epirubicin could activate ataxia-telangiectasia mutated (ATM) that promotes E2F activity and FOXM1 expression [83]. In addition, a study by de Olano et al. showed that the mechanism of epirubicin resistance was mediated by activation of mitogen-activated protein kinase-activated protein kinase 2 leading to increased phosphorylation of transcription factor E2F1 at Ser-364 resulting in increased FOXM1 expression [84]. Khongkow et al. demonstrated that FOXM1 reduced senescence induced by epirubicin, by increasing expression of NBS1 leading to enhanced homologous recombination DNA repair [85]. Another work by Khongkow et al. postulated that resistance to paclitaxel, a tubulin targeting agent, can be mediated by FOXM1 through enhancement of promoter activity of transcriptional activity of KIF20A [86]. Both FOXM1 and KIF20A are critical for normal formation of mitotic spindle and thus could interfere with paclitaxel activity. Nestal de Moraes et al. [43] demonstrated that resistance to epirubicin, docetaxel, and paclitaxel was associated with activation of XIAP and survivin by direct interaction of FOXM1 with promoters of these antiapoptotic genes.

## 6. Conclusion

Forkhead box M1 plays a significant role in breast carcinogenesis, disease progression to metastatic stage, and development of resistance to subsequent cancer therapy, and thus it could be an attractive target for therapeutic interventions in this malignancy. However, due to incomplete understanding of the biology of the FOX family of transcription factors that has complex regulatory mechanisms, it has been a challenge to develop a drug that functions specifically as a FOXM1 inhibitor [87]. In addition, druggability of FOXM1 has been taxing because of lack of substrate binding pockets and hydrophobic surfaces [88]. A recent discovery through high throughput fluorescence polarization assay of a novel small molecule that specifically inhibits FOXM1 interaction with DNA may be a first breakthrough in the design of selective inhibitors of this transcription factor [89]. Once FOXM1 targeted agents are available, appropriate clinical testing in different breast cancer subtypes is warranted, most likely in a biomarker-driven setting. At present, however, there is no clear biomarker developed to assess sensitivity of breast cancer to FOXM1 inhibition. This could be a goal of early pilot studies, especially in the most challenging breast cancer subtype, triple negative disease.
